# Effects of Salicylic Acid on Physiological Responses of Pepper Plants Pre-Subjected to Drought under Rehydration Conditions

**DOI:** 10.3390/plants13192805

**Published:** 2024-10-07

**Authors:** Fabrício Custódio de Moura Gonçalves, Luís Paulo Benetti Mantoan, Carla Verônica Corrêa, Nathália de Souza Parreiras, Luiz Fernando Rolim de Almeida, Elizabeth Orika Ono, João Domingos Rodrigues, Renato de Mello Prado, Carmen Sílvia Fernandes Boaro

**Affiliations:** 1 Department of Horticulture, Faculty of Agricultural Sciences, São Paulo State University (UNESP), Campus de Botucatu, Avenida Universitária, 3780 Altos do Paraíso, Botucatu 18610-034, Brazil; cvcorrea1509@gmail.com (C.V.C.); nathaliaparreiras@ig.com.br (N.d.S.P.); 2 Department of Biodiversity and Biostatistics, Institute of Biosciences, São Paulo State University (UNESP), Campus de Botucatu, R. Prof. Dr. Antônio Celso Wagner Zanin, 250 Distrito de Rubião Junior, Botucatu 18618-689, Brazil; luismantoan@gmail.com (L.P.B.M.); luiz.rolim@unesp.br (L.F.R.d.A.); elizabeth.o.ono@unesp.br (E.O.O.); joao.domingos@unesp.br (J.D.R.); carmen.boaro@unesp.br (C.S.F.B.); 3 Department of Soils and Fertilizers, Faculty of Agricultural and Veterinary Sciences, São Paulo State University (UNESP), Campus Jaboticabal, Prof. Paulo Donato Castellane s/n, Jaboticabal 14884-900, Brazil; rm.prado@unesp.br

**Keywords:** vegetal regulator, water deficit, photosynthetic pigments, stomatal conductance, chlorophyll *a* fluorescence, hydrogen peroxide

## Abstract

*Capsicum annuum* L. has worldwide distribution, but drought has limited its production. There is a lack of research to better understand how this species copes with drought stress, whether it is reversible, and the effects of mitigating agents such as salicylic acid (SA). Therefore, this study aimed to understand the mechanisms of action of SA and rehydration on the physiology of pepper plants grown under drought conditions. The factorial scheme adopted was 3 × 4, with three water regimes (irrigation, drought, and rehydration) and four SA concentrations, namely: 0 (control), 0.5, 1, and 1.5 mM. This study evaluated leaf water percentage, water potential of shoots, chlorophylls (*a* and *b*), carotenoids, stomatal conductance, chlorophyll *a* fluorescence, and hydrogen peroxide (H_2_O_2_) concentration at different times of day, water conditions (irrigation, drought, and rehydration), and SA applications (without the addition of a regulator (0) and with the addition of SA at concentrations equal to 0.5, 1, and 1.5 mM). In general, exogenous SA application increased stomatal conductance (*gs*) responses and modified the fluorescence parameters (ΦPSII, qP, ETR, NPQ, D, and E) of sweet pepper plants subjected to drought followed by rehydration. It was found that the use of SA, especially at concentrations of 1 mM in combination with rehydration, modulates *gs*, which is reflected in a higher electron transport rate. This, along with the production of photosynthetic pigments, suggests that H_2_O_2_ did not cause membrane damage, thereby mitigating the water deficit in pepper plants. Plants under drought conditions and rehydration with foliar SA application at concentrations of 1 mM demonstrated protection against damage resulting from water stress. Focusing on sustainable productivity, foliar SA application of 1 mM could be recommended as a technique to overcome the adverse effects of water stress on pepper plants cultivated in arid and semi-arid regions.

## 1. Introduction

Water deficit is the second most damaging abiotic stress in agricultural crops worldwide. Temporary water deficit influences physiological, biochemical, metabolic, and morphological processes, interfering with plant growth and development and consequently plant productivity [1,2]. Water deficiency is a complex process, and plants respond to stress through mechanisms that avoid or lead to adaptation to these effects [3,4].

In plants under water deficit, the formation of chlorophyll can be inhibited due to the increased production of hydrogen peroxide, which causes damage to membranes, degradation of pigments [5], and nutrient uptake. In periods of drought, lack of water can alter the photosynthetic activities of plants [6], modifying stomatal conductance (*gs*) and restricting the entry of CO_2_ into the substomatal chamber, decreasing sugar production, and affecting the plant’s source–sink relationship and overall development [7,8].

The reduction in CO_2_ assimilation caused by stomatal closure contributes to the imbalance between photochemical activity in photosystem II (PSII) and the requirement for electrons for photosynthesis, promoting photoinhibitory damage to the PSII reaction center [9,10]. This causes the deviation of absorbed light to other processes, such as thermal dissipation to protect the photosynthetic apparatus [11,12], especially under stress conditions due to water scarcity.

In this scenario, it is imperative to overcome the deleterious effects of drought stress on cultivated plants by adopting appropriate technologies. Under water stress conditions, exogenous resistance inducers have been applied, which act in plant protection, improving plant defense mechanisms. Among defense inducers, salicylic acid (SA) stands out, which is an endogenous signaling molecule involved in physiological processes and in the induction of biosynthesis reactions for the development of protection systems [13,14,15]. By lowering cellular lipid peroxidation and hydrogen peroxide accumulation, SA application to plants with insufficient water lowers cell membrane damage in leaves due to the increased activity of antioxidant enzymes such as catalase, superoxide dismutase, peroxidase, and guaiacol peroxidase [16], that help neutralize free radicals and reduce oxidative stress associated with water stress. In addition, SA increases chlorophyll and carotenoid content [17].

Thus, SA has been used to modulate the physiological and biochemical responses of plants subjected to drought [18,19]. Among physiological responses, SA can influence the expression of genes related to photosynthesis, including those encoding proteins of the photosynthetic reaction complex and components of the Calvin cycle, interfering both in the activity of the ribulose-1,5-bisphosphate carboxylase oxygenase (RuBP) [15,20], and by regulating transpiration under drought conditions, which reduces water loss and maintains turgor, leading to maintenance of photosynthesis and productivity [21,22].

Therefore, the attenuating effect of SA under drought conditions may be associated with its role in photosynthesis [23,24] and in stomatal regulation [25], which, in turn, act as pathways to eliminate reactive oxygen species and provide protection to the photosynthetic apparatus under stress conditions [26,27]. In certain circumstances, SA can antagonize the effects of abscisic acid (ABA), a plant hormone that typically induces stomatal closure in response to water stress. This can result in higher stomatal conductance and transpiration rate [28].

Pepper (*Capsicum annuum* L.) is a warm-season crop, and water deficit is considered a major issue in pepper cultivation, negatively affecting morphophysiological characteristics, yield, and quality [29,30].

*C. annuum* L. is an economically important horticultural plant belonging to the Solanaceae family. Peppers are not only valuable as a food crop but also for their medicinal properties. Specifically, hot peppers have anti-inflammatory and analgesic properties due to the presence of capsaicin. They also contain essential nutrients, including vitamins C and E, beta-carotene, phenolic compounds, flavonoids, folate, protein, mineral elements, and fiber. As such, peppers are considered a potent source of antioxidants, contributing to improving the immune system [31,32].

In recent years, the harvested area and production of peppers have increased significantly. Plants must develop complex mechanisms to cope with various stresses, especially under the current global environmental challenges [33]. Among these, the increasing frequency of droughts due to climate change has emerged as a critical stress factor. More research is needed to understand how this species responds to water deficits in the presence of defense inducers. By understanding the SA mechanisms of action and the effects on pepper plants’ physiological responses to stress, farmers can develop more effective strategies to mitigate the negative impacts of water stress.

In this context, the application of SA may represent a promising approach for inducing drought resistance mechanisms in peppers and several other crops of agricultural interest, including vegetables, which are generally sensitive to water stress [28,34]. SA plays a crucial role in protecting horticultural crops, increasing productivity, and maintaining food security in the face of adverse environmental conditions by mitigating the harmful effects of water stress [28]. Foliar SA application is, therefore, a viable strategy for improving productivity and crop resilience, especially in arid and semi-arid regions [35,36].

‘Melina’ hybrid pepper, suited for cultivation in tropical regions, is highly accepted by producers. However, it is vulnerable to adverse weather conditions, such as cycles of drought followed by rain, which can prevent plant recovery due to successive stress cycles. Therefore, it was hypothesized that rehydration combined with exogenous SA application strengthens the physiological responses of pepper plants, promoting carbon dioxide assimilation and pigment production, reducing damage to the photosynthetic apparatus, and enhancing plant defense during drought periods [37,38].

Thus, it is essential to investigate whether foliar SA application to drought-stressed pepper plants, followed by rehydration, improves stress tolerance and enhances recovery efficiency. This study offers a new perspective on the interaction between SA and rehydration, encouraging the performance of research and biotechnological advancements focused on sustainable agriculture.

The hypothesis of this research is that exogenous SA application, combined with rehydration, improves shoot water potential, photosynthetic pigment concentration, stomatal conductance, and chlorophyll a fluorescence throughout the day. This effect is crucial for mitigating the production of hydrogen peroxide in young sweet pepper (‘Melina’ hybrid *C. annuum*) plants subjected to drought.

If this hypothesis is confirmed, it will propose strategies for optimizing the use of SA to combat water deficits, with global implications given the increasing frequency of droughts due to climate change, which affects pepper production in various growing regions.

This study investigated the acclimation of chlorophyll *a* fluorescence, photosynthetic pigments, stomatal conductance, and water potential of pepper plants during rehydration after a period of water scarcity in the presence and absence of salicylic acid.

## 2. Results

### 2.1. Effects of Treatments on Relative Leaf Water Content, Plant Water Potential, Chlorophyll Content (a and b), and Carotenoids

The water deficit in plants was quantified by the relative leaf water content (RWC), expressed as a percentage (Supplementary Table S1), and by the plant water potential in kilopascals (Supplementary Table S2). Leaf RWC varied throughout the day, with lower percentages observed at times of higher temperatures (12:00 and 14:00 h) in the drought treatment (Supplementary Table S1 and Supplementary Figure S1A). During this period (12:00 and 14:00 h), the vapor pressure deficit (VPD) was also high, 7 days after treatments (DAT) (Supplementary Figure S1C).

**Figure 1:**
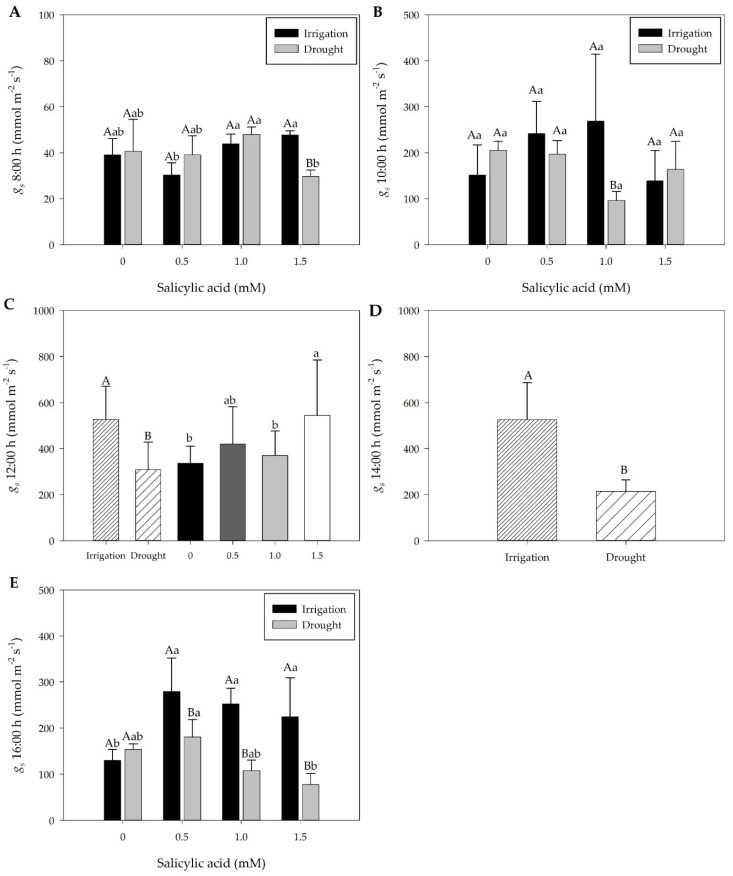
Stomatal conductance (*gs*) in mmol m^−2^s^−1^ throughout the day, 8:00 h (**A**), 10:00 h (**B**), 12:00 (**C**) h, 14:00 h (**D**), and 16:00 h (**E**) at seven days after treatments (7 DAT). Black bars represent irrigation treatment; white bars represent irrigation without treatment. Letters show difference between treatments by the 5% Tukey’s test. Values represent means ± standard deviation of four replicates. Capital letters compare means of water conditions, and lowercase letters compare means of salicylic acid concentrations.

In general, drought-treated plants at 7 DAT showed a reduction in RWC over time, with values of 73.6% at 8:00 h and 63.6% at 16:00 h (Supplementary Table S1). Under these conditions, the stomatal conductance (*gs*) values, especially between 12:00 and 14:00 h, were lower (Figure 1C,D). Despite the constant decrease in RWC (Supplementary Table S1), the plant water potential, as expected, maintained the relative water content (kPa) (Supplementary Table S2). At 16:00 h, drought-treated plants recovered their RWC, similar to control plants (Supplementary Table S1).

Upon rehydration, at 12 days after treatments (DAT), with the increase in soil water content, RWC reached values similar to those of irrigated plants at the different evaluation times (Supplementary Table S1). The RWC percentage in plants subjected to the irrigation treatment and the rehydration treatment of plants subjected to drought 12 DAT (5 days after rehydration) did not differ either in relation to time or salicylic acid (SA) application (Supplementary Table S1). The plant water potential did not vary in plants in the irrigation and drought treatments, with no effect of SA application at 7 and 12 DAT (Supplementary Table S2).

Chlorophylls *a* and *b* and carotenoids did not differ in plants not subjected to drought at 7 DAT. However, when the highest SA concentration (1.5 mM) was applied, no reduction in chlorophyll *a* and carotenoid contents was observed in comparison to control plants (Supplementary Table S2).

Plants in irrigation and rehydration treatments and those submitted to SA concentrations showed no difference in water potential, chlorophylls *a* and *b*, or carotenoids at 12 DAT (Supplementary Table S2).

### 2.2. Effect of Treatments on Stomatal Conductance

The stomatal conductance (*gs*) of irrigated plants was higher than that of drought-treated plants (Figure 1), a result also observed for leaf RWC (Supplementary Table S1). Under drought conditions, the constant decrease in soil water content led to a reduction in leaf RWC from 74.2% to 70.2% at 12:00 h and from 70.1% to 64.9% at 14:00 h (Supplementary Table S1). Under these conditions, there was a reduction in relative humidity and *gs* 7 days after treatments (DAT) (Figure 1C,D, and Supplementary Figure S1B).

**Figure 2:**
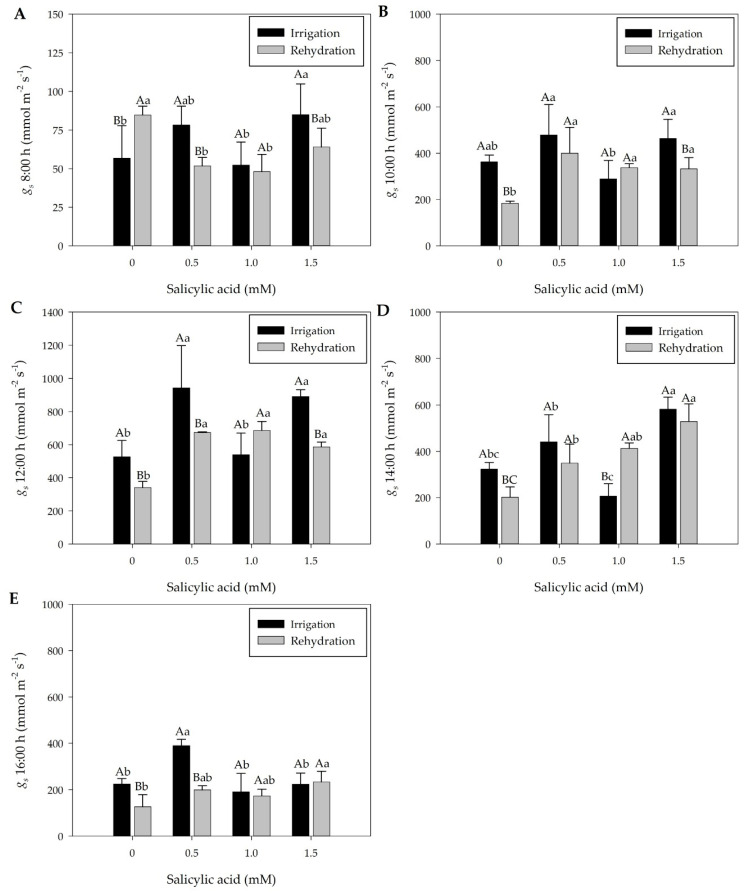
Stomatal conductance (*gs*) in mmol m^−2^s^−1^ throughout the day, 8:00 h (**A**), 10:00 h (**B**), 12:00 h (**C**), 14:00 h (**D**), and 16:00 h (**E**) at twelve days after treatments (12 DAT). Black bars represent irrigation treatment; white bars represent rehydration treatment. Letters show the difference between treatments by the 5% Tukey’s test. Values represent means ± standard deviation of four replicates. Capital letters compare means of water conditions, and lowercase letters compare means of salicylic acid concentrations.

The *gs* values of plants under drought showed significant reduction in comparison with irrigated plants, since, under water deficit conditions, the low water availability contributes to stomatal closure and reduction of *gs* values (Figure 1A–E). Subsequently, upon rehydration, the *gs* of plants increased but did not reach the same values as irrigated plants (Figure 2A–E), while leaf RWC did not vary (Supplementary Table S1).

In Figure 1C,D, we observe a lack of interaction between the water regimes (irrigation vs. drought) and the foliar application of SA. These figures present separate effects of water regime and SA treatment on *gs*, without any synergistic or antagonistic interaction between the factors. Specifically, the *gs* values in irrigated plants remain consistently higher than those under drought, regardless of SA application. This indicates that while SA does exert a positive effect on *gs*, the water regime remains the dominant factor controlling stomatal behavior under stress conditions. The reason these figures exhibit a different pattern compared to others in the study lies in the nature of *gs* regulation under drought stress. Unlike other parameters, which may show combined effects of water status and SA, stomatal conductance is largely regulated by water availability, which overrides potential SA-induced modulations.

At 7 DAT, at 8:00 and 16:00 h, plants subjected to 1.5 mM SA and drought showed lower *gs* values (Figure 1A,E). During these times, temperature and VPD were lower (Supplementary Figure S1A,C). At 10:00 h, plants with and without SA exhibited *gs* values similar to those of irrigated plants (Figure 1B). At 12:00 h, there was no interaction between irrigation and drought with SA, and plants treated with 1.5 mM SA under these conditions (higher temperature and VPD) showed higher *gs* values (Figure 1C and Supplementary Figure S1A,C). At 14:00 h, results showed only the effect of the watering regime, with irrigated plants exhibiting higher *gs* values (Figure 1D).

Specifically, the models for Figure 1C,D do not reflect interaction but instead show distinct responses to water regimes and SA concentrations in isolation. This distinction highlights the specific physiological role of *gs* in controlling water loss through transpiration, which is tightly coupled to plant water status rather than to external applications of plant regulators. Thus, the models presented in Figure 1C,D help emphasize that under severe drought stress, the physical limitation of water availability is the primary driver of stomatal behavior, independent of hormonal regulation by exogenous SA.

At 12 days after treatments (DAT), rehydrated plants showed significant differences in *gs* values compared to irrigated plants at 8:00, 10:00, 12:00, 14:00, and 16:00 h, generally exhibiting lower values in rehydrated plants (Figure 2A–E). Additionally, it was observed that rehydrated plants at 12:00 h showed higher *gs* values (average 571.5 mmol m^−2^s^−1^) compared to drought-treated plants (average 309 mmol m^−2^s^−1^) (Figure 1 and Figure 2). At 12:00 h, temperature and VPD were high (Supplementary Figure S1A,C). Furthermore, at 12:00 h, RWC was lower in drought-treated plants, increasing in rehydrated plants (Supplementary Table S1).

The *gs* values varied in plants subjected to different SA concentrations, both in irrigated plants and in those subjected to rehydration, allowing us to recommend the SA concentration of 1 mM that contributes to the increase in *gs* values (Figure 2B–D) under the rehydration treatment of pepper plants at 12 DAT. Plants subjected to 0.5 mM of SA and rehydrated plants did not change *gs* values (Figure 2), and showed higher hydrogen peroxide concentration during rehydration, signaling stress and activation of the antioxidant system, without showing variation in the *gs* concentration, photosynthetic pigments, or RWC (Supplementary Tables S1 and S2). At 16:00 h, the application of 1.5 mM of SA increases *gs* values, with a variation of photochemical quenching values (qP) (Figure 2E and Figure 4C).

### 2.3. Effects of Treatments on Chlorophyll Fluorescence

**Figure 3:**
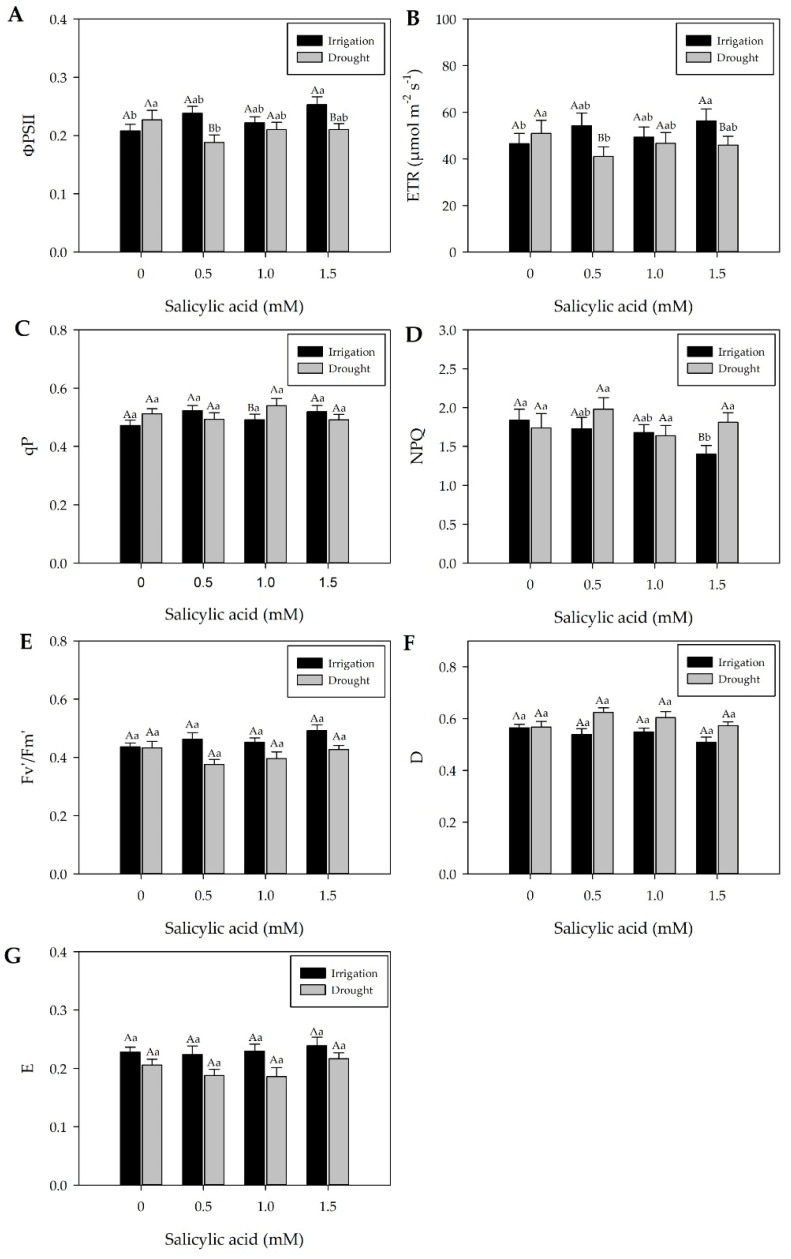
Effective quantum yield—ΦPSII (**A**), electron transport efficiency—ETR (**B**), photochemical quenching—qP (**C**), non-photochemical quenching—NPQ (**D**), maximum quantum yield—Fv′/Fm′ (**E**), heat dissipation—D (**F**), and dissipation of unused energy—E (**G**) seven days after treatments (7 DAT), determined at 12:00 h. Black bars represent irrigation treatment; white bars represent drought treatment. Letters show the difference between treatments by the 5% Tukey’s test. Values represent means ± standard deviation of four replicates. Capital letters compare means of water conditions, and lowercase letters compare means of salicylic acid concentrations.

Chlorophyll fluorescence and stomatal conductance (*gs*) decreased gradually with water stress intensity and duration (Figure 1 and Figure 3).

At 7 days after treatments (DAT) (5 days before rehydration), chlorophyll *a* fluorescence can be observed in Figure 3A–G. Generally, effective quantum yield (ΦPSII) and electron transport rate (ETR) showed similar variations in plants subjected to drought and irrigated plants (Figure 3A,B). Both ΦPSII and ETR in drought-treated plants decreased due to water deficit stress compared to irrigated plants (Figure 3A,B). Similarly, *gs*, chlorophyll *a*, carotenoid contents, and leaf RWC also decreased under drought conditions (Figure 1 and Supplementary Tables S1 and S2). The non-photochemical quenching (NPQ) and the photochemical quenching (qP) values of plants subjected to drought were higher compared to those of irrigated plants (Figure 3C,D). Plants under drought conditions revealed lower dissipation of unused energy (E) and a lower RWC percentage (Figure 3G and Supplementary Table S1).

The maximum quantum yield (Fv′/Fm′) of sweet pepper plants at 7 DAT did not differ in relation to the irrigation or drought treatments. The Fv′/Fm′ ratio was used to indicate photoinhibitory damage in plants subjected to drought. It was observed that the water suspension to which sweet pepper plants were submitted was not enough to cause photoinhibitory damage (Figure 3E). The same behavior was observed for both heat dissipation by xanthophylls (D) and dissipation of unused energy (E) (Figure 3F,G).

In general, effective quantum yield (ΦPSII), electron transport (ETR), maximum quantum yield (Fv′/Fm′), photochemical quenching (qP), non-photochemical quenching (NPQ), heat dissipation (D), and energy dissipation (E) of plants subjected to drought showed the same behavior as irrigated plants at SA concentrations of 1 mM (Figure 3A–G).

Although irrigation increased ΦPSII and ETR values, exogenous SA application does not modify ΦPSII and ETR of plants subjected to drought and irrigated conditions (Figure 3A,B). It should be noted that plants subjected to drought without SA exhibit elevated ΦPSII and ETR values (Figure 3A,B). As for qP, there are fluctuations in plant responses across watering regimes. However, the presence or absence of SA in both watering regimes does not modify qP (Figure 3C).

The NPQ of plants subjected to drought 7 DAT showed a reduction in irrigated plants with an SA concentration of 1.5 mM, resulting in less energy dissipation in the form of heat in plants under irrigation (Figure 3D,F). SA did not modify qP, Fv′/Fm′, D, and E (Figure 3C,E–G).

**Figure 4:**
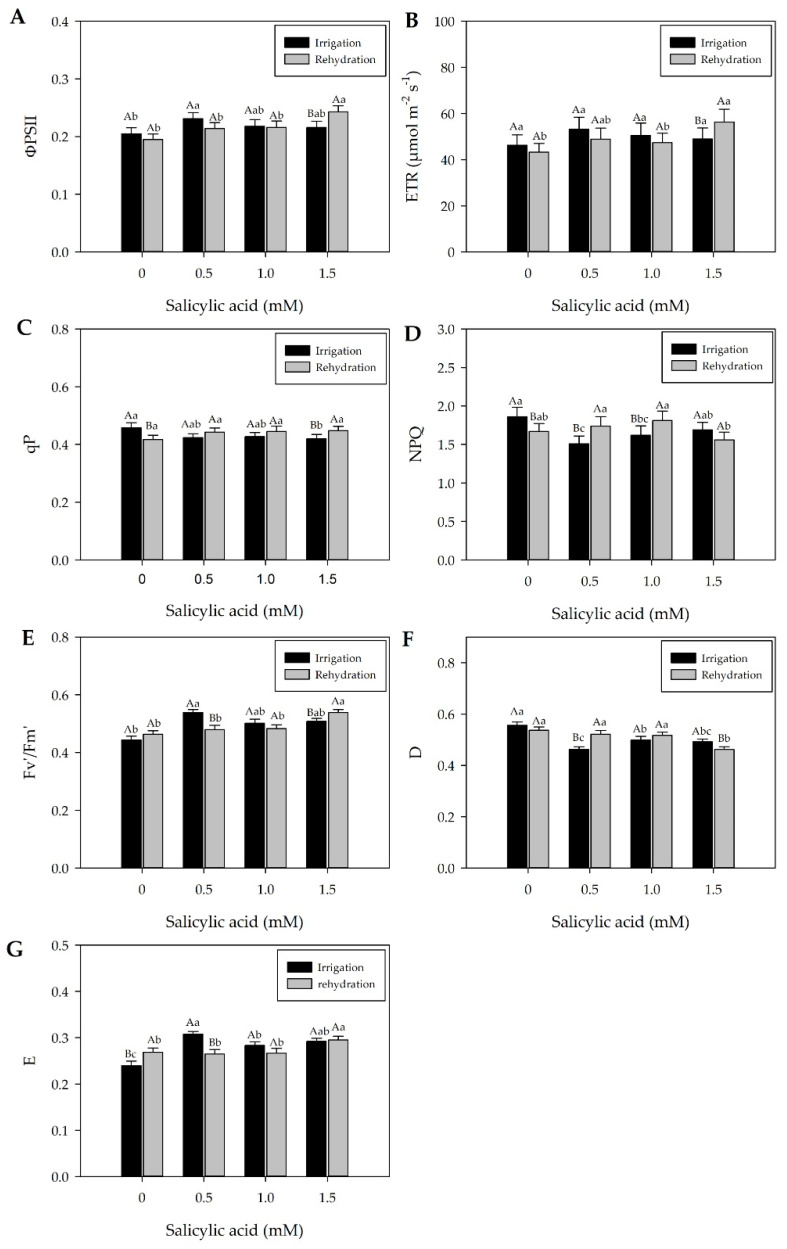
Effective quantum yield—ΦPSII (**A**), electron transport efficiency—ETR (**B**), photochemical quenching—qP (**C**), non-photochemical quenching—NPQ (**D**), maximum quantum yield—Fv′/Fm′ (**E**), heat dissipation—D (**F**), and dissipation of unused energy—E (**G**) twelve days after treatments (12 DAT), determined at 12:00 h. Black bars represent irrigation treatment; white bars represent rehydration treatment. Letters show the difference between treatments by the 5% Tukey’s test. Values represent means ± standard deviation of four replicates. Capital letters compare means of water conditions, and lowercase letters compare means of salicylic acid concentrations.

During drought, gas exchange rates (*gs*) and RWC decreased (Figure 1C,D, and Supplementary Table S1). However, after 5 days of rehydration (12 DAT), leaf RWC recovered to levels similar to irrigated plants, with improved *gs* and no changes in leaf pigment content (Supplementary Tables S1 and S2, Figure 2). At the same time (12 DAT), rehydrated and irrigated plants generally showed similar ΦPSII and ETR values (Figure 4A,B), except when 1.5 mM of SA was applied. Rehydrated plants exhibited higher qP, NPQ, and D values (Figure 4C,D,F). Overall, in rehydration, young pepper plants (Hib. ‘Melina’) showed a positive correlation between chlorophyll *a* fluorescence and *gs* (Figure 2 and Figure 4).

In general, there was no statistical difference in the Fv′/Fm′ ratio between irrigation and rehydration plants with values ranging, respectively, from 0.497 to 0.490, possibly demonstrating a non-stressful condition since the Fv′/Fm′ ratio is used as an indicator of stress conditions on the photosynthetic apparatus (Figure 4E).

Plants subjected to irrigation with the lowest SA concentration (0.5 mM) showed higher ΦPSII, while those with 1 mM of SA exhibited higher ETR (Figure 4A,B).

The qP value showed reduction in irrigation plants subjected to the highest SA concentration (1.5 mM). Even at this concentration, rehydration plants increased qP values (Figure 4C). This result interferes with the photosynthetic carbon metabolism. Based on the results above, it could be inferred that the SA concentration of 1.5 mM can help to successfully complete the electron transport (Figure 4B); however, the D and E values of these plants did not show variations (Figure 4F,G).

Irrigation plants showed reduction in NPQ, with SA concentrations of 0.5 and 1 Mm (Figure 4D). Higher D values were found when 0.5 and 1.0 mM of SA were applied during rehydration (Figure 4F). Application of 0.5 mM of SA also resulted in greater energy accumulation in the photosystems (E) and Fv′/Fm′ of irrigated plants (Figure 4G,E).

### 2.4. Effect of Treatments on Hydrogen Peroxide

**Figure 5:**
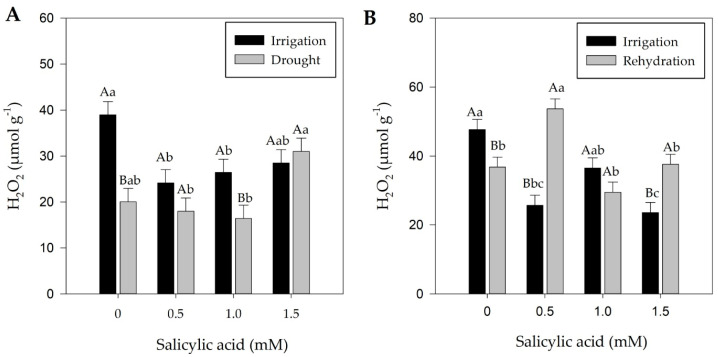
Hydrogen peroxide concentrations seven (**A**) and twelve (**B**) days after treatments. Black bars represent irrigation treatment; white bars represent rehydration treatment. Letters show the difference between treatments by the 5% Tukey’s test. Values represent means ± standard deviation of four replicates. Capital letters compare means of water conditions, and lowercase letters compare means of salicylic acid concentrations.

Temporary drought caused a reduction in hydrogen peroxide (H_2_O_2_) production, while plants without water deficit showed higher H_2_O_2_ levels (Figure 5A) at 7 days after treatments (DAT). During drought, plants exhibited reduced stomatal conductance (*gs*) and, overall, chlorophyll *a* fluorescence parameters (Figure 1 and Figure 3), as well as a decrease in leaf relative water content (RWC) from 73.6% at 8:00 h to 63.6% at 16:00 h (Supplementary Table S1).

At 12 days after treatments (DAT), rehydrated bell pepper plants showed higher H_2_O_2_ concentrations compared to irrigated plants (Figure 5B), except at concentrations of 1 mM and without SA. RWC, plant water potential, chlorophyll *a* and *b* content, and carotenoid levels did not show differences (Supplementary Tables S1 and S2), indicating recovery. However, although *gs* values partially recovered (Figure 2), there were no changes in ΦPSII and ETR, except when 1.5 mM of SA was applied (Figure 4A,B).

The H_2_O_2_ content did not differ between plants under drought and those sprayed with SA at 7 DAT (Figure 5A), consistent with plant water potential, pigment levels, Fv′/Fm′, D, and E (Supplementary Table S2 and Figure 3E–G), which also showed no differences.

Rehydration (12 DAT) plants showed higher H_2_O_2_ concentrations at SA concentrations of 0.5 and 1.5 mM (Figure 5B). When exogenously applied, SA of 0.5 mM acts by raising the level of reactive oxygen species under rehydration conditions. These factors contribute to the activation and regulation of antioxidant enzymes (POD, CAT, and SOD) to maintain the H_2_O_2_ balance. Conversely, the highest SA concentration, 1.5 mM, may result in stress (Figure 5B), revealing lower levels of chlorophyll *a* and carotenoids (Supplementary Table S2).

## 3. Discussion

### 3.1. Effect of Drought and Rehydration on Irrigated Plants Cultivated without Salicylic Acid

The response of plants subjected to water deficit varies according to the environment and genotype. Research on mechanisms of bell peppers (*Capsicum annuum* L.) to cope with water deficits remains poorly understood. This is a cause for concern given the advancement of climate change and the occurrence of drought in different countries that cultivate this species.

The drop in the relative leaf water content (RWC) from 74.2% to 70.2% at 12:00 h and from 70.1% to 64.9% at 14:00 h for pepper plants under drought reflected in the decrease of stomatal conductance (*gs*) values during these periods throughout the day (Supplementary Table S1 and Figure 1), 7 days after treatments (DAT). In addition to suspending irrigation, environmental variation must be taken into account, which influences the water potential gradient between soil, plant, and atmosphere (Supplementary Figure S1A–C). Thus, the lower soil water availability hinders its flow via the xylem, resulting in less water in leaves, especially at warmer times and with lower relative humidity, as observed between 12:00 and 14:00 h. Additionally, the increase in vapor pressure deficit (VPD) and temperature at 12:00 h may contribute to damage to membrane systems, influencing water absorption by roots (Supplementary Table S1 and Supplementary Figure S1A,C). Leaves of plants subjected to drought close their stomata to reduce water loss through transpiration, which could reduce CO_2_ assimilation (Figure 1). If this condition persists, it can affect normal cell function and plant growth.

Despite the reduction in RWC under drought conditions between 12:00 and 14:00 h, plants recovered their leaf water potential by 16:00 h, which became equal to that of irrigated plants at 7 DAT. The lower *gs* at this time may have contributed to the recovery of RWC (Supplementary Table S1 and Figure 1E. This result demonstrates the capacity of pepper plants to recover RWC by the end of the day and acclimatize. It should be noted that the plant water potential was also not influenced by the watering regime (Supplementary Table S2). Sweet pepper plants kept with higher water potential in soil and leaves (irrigation treatment) showed higher *gs* values when compared to those subjected to a gradual increase in water stress (Supplementary Table S1 and Figure 1).

The lower *gs* values of plants under drought stress at 7 DAT may be related to the decrease in chlorophyll *a* fluorescence (Figure 1 and Figure 3), as indicated by the lower electron transport rate (ETR) and effective quantum yield (ΦPSII) (Figure 3A,B). The reduction in leaf RWC from 73.6% at 8:00 h to 63.6% at 16:00 h in drought-stressed plants (Supplementary Table S1) could intensify oxidative damage to lipids and alterations in electron transport (Figure 3B and Figure 5A). Under drought conditions, electron transport inhibition can compromise adenosine triphosphate (ATP) and nicotinamide adenine dinucleotide phosphate (NADPH) synthesis in the chloroplast [39], leading to a reduction in ΦPSII. This reduction in ΦPSII under low activity results in an imbalance between electron generation and utilization. As a consequence, the inability to convert absorbed light energy by chlorophyll into electrochemical energy leads to the production of singlet oxygen [40], which can damage the photosynthetic apparatus and even suppress enzymatic activities [41].

In summary, SA exerts a multifaceted influence on plant response to drought, acting through various molecular and biochemical pathways to modulate key physiological processes such as *gs*, transpiration, and photosynthetic efficiency.

During rehydration, at 12 days after treatments (DAT), plants showed elevated *gs* values, possibly due to the maintenance of RWC in leaves (Supplementary Table S1 and Figure 2). Although plants exhibited an increase in *gs* values, this increase did not reach the level of irrigated plants (Figure 2), suggesting that the recovery of *gs* values in pepper plants may take longer. However, at 12:00 h, the *gs* recovery of rehydrated plants was higher (average of 571,5 mmol m^−2^ s^−1^), compared to drought-stressed plants with an average of 309 mmol m^−2^ s^−1^ (Figure 1C and Figure 2C).

After rehydration, pepper plants show elevated ETR values (Figure 4B), which can contribute to CO_2_ assimilation. These results suggest that the hybrid ‘Melina’ pepper exhibited adjustments in gas exchange and possible short-term photosynthetic acclimation to water deficiency over five days followed by rehydration (Supplementary Table S2 and Figure 2B–D). At 12 DAT, plants showed elevated non-photochemical quenching (NPQ) and photochemical quenching (qP) values, suggesting signaling of photoprotective responses in the species in the presence of water (Figure 4C,D).

During rehydration, the partial recovery of *gs* values (Figure 2) may have contributed to the non-detection of damage to ΦPSII (Figure 4A). ΦPSII is widely used to detect disturbances induced by stress in the photosynthetic apparatus [11,12,13,14,15,16,17,18,19,20,21,22,23,24,25,26,27,28,29,30,31,32,33,34,35,36,37,38,39,40,41,42]. Interestingly, when pepper plants from drought conditions were subjected to rehydration, they showed higher H_2_O_2_ values compared to irrigated plants (Figure 5B), indicating responses of the species’ enzymatic defense system.

### 3.2. Effect of Salicylic Acid on Mitigating the Effects of Drought and Rehydration Compared to Irrigated Conditions

It has been evidenced that drought impairs the physiological aspects of bell peppers and highlights the importance of plant rehydration. However, there is a lack of research in this species to determine whether foliar salicylic acid (SA) can in fact enhance drought tolerance and the mechanisms involved. This information is highly relevant because SA has relatively low costs when applied in leaves, which requires small amounts and poses no risk to the environment, which could strengthen the cultivation of this species on sustainable bases.

Exogenous SA application appears to play a crucial role in improving plant gas exchange, as indicated by the increase in *gs* values (Figure 1C) at 7 DAT. This observation is consistent with findings in the literature suggesting that SA may modulate the activity of enzymes such as ribulose-1,5-bisphosphate carboxylase/oxygenase (RuBP), indirectly affecting water use efficiency [43]. However, the use of SA had no effect on RWC and plant water potential (Supplementary Tables S1 and S2). Foliar SA treatment at the highest concentration (1.5 mM) was the best treatment to increase leaf relative water content (83.3%) of pepper plants [44].

At 7 DAT, the results of this study highlight that specific SA concentrations, namely 0.5 and 1 mM, were effective in maintaining the *gs* values in pepper plants subjected to drought (Figure 1A,B,E, and Figure 2B–D). Xu and Tian [45] reported that SA acts as a secondary messenger involved in signal transduction in response to stress, inducing reactions that promote plant protection. González-Villagra et al. [46] reinforce that plants treated with SA generally have higher relative water content and higher activity of the ribulose-1,5-bisphosphate carboxylase/oxygenase enzyme. Both the RWC and the plant water potential did not differ between irrigation and rehydration plants subjected to SA concentrations, demonstrating that the recovery of plant hydration does not depend on the use of plant regulators (Supplementary Tables S1 and S2). In general, ΦPSII, ETR, maximum quantum yield (F_V_′/Fm′), qP, NPQ, heat dissipation (D), and dissipation of unused energy (E) of plants subjected to drought showed the same behavior as irrigated plants at SA concentrations of 1 mM (Figure 3A–G). Exogenous SA application resulted in a high photosynthetic rate and alleviated drought-induced effects on net photosynthesis and efficiency of ΦPSII [47].

However, higher SA concentrations (1.5 mM) may lead to negative effects, such as reduction in photosynthesis (Figure 1E) and increased oxidative stress, as evidenced by decreased levels of chlorophyll *a* and carotenoids, regardless of stress (Supplementary Table S2). This highlights the importance of determining ideal SA concentrations to maximize its beneficial effects and minimize any adverse effects. As high SA concentrations (1.5 mM) can harm physiological processes, the application of plant regulators at low concentrations is recommended, which can promote the recovery of plants subjected to stress [48,49].

Research shows that beneficial effects under stress conditions are possible at SA concentrations ranging from 0.05 to 0.5 mM [48]. Waseem et al. [50] reported that exogenous SA application can induce resistance to drought in wheat plants. Foliar SA application improves the photosynthetic rate of pepper plants by increasing leaf chlorophyll content and RWC and regulating *gs* levels [36,51]. However, this result is different from that obtained by Shan et al. [48], who observed that under drought conditions, photosynthetic pigments decreased considerably while SA increased the carotenoid content in basil plants, and according to Ghahremani et al. [44], foliar SA application at 1.5 mM in pepper plants was the best treatment to increase leaf *gs* under water stress conditions (367.4 mM m^−2^ s^−1^). They also observed that the highest leaf chlorophyll content was recorded in pepper plants sprayed with SA at 1.5 mM. Although the beneficial effects of SA are described in literature, plant responses to the exogenous application of this resistance inducer are still controversial [34,52] and the authors reinforce that exogenous SA application can negatively influence the plant’s defense processes, especially at high concentrations (Supplementary Table S2 and Figure 1E). However, at 7 DAT, foliar SA increased NPQ in drought-stressed plants, and 1.5 mM of SA is indicative of signaling of the photoprotective capacity of this concentration (Figure 3D).

In general, foliar SA application of 1 mM plays a role in maintaining the activity of ΦPSII under drought conditions in pepper plants, which could alleviate the effects of reactive oxygen species, thereby maintaining the stability of components of the ΦPSII reaction center and electron transport (Figure 3A and Figure 5A).

During rehydration at 12 DAT, SA treatments were more effective in increasing *gs* values than the watering regimes (Figure 2), as observed at 10:00, 12:00, and 14:00 h. An SA concentration of 1 mM contributes to increase *gs* values (Figure 2B–D) under rehydration pepper plants. At 12 DAT, 1 mM of SA contributed to increasing ETR values (Figure 4B). Aires et al. [21] verified that *gs* also increased with SA concentrations of 1.4 and 1.1 mM under water stress in tomato plants.

During rehydration, SA contributed to maintaining Fv′/Fm′ ratio (Figure 4E), which may have helped reduce damage to the photosynthetic apparatus. In tomato plants subjected to drought, an SA application of 10^−3^ M resulted in a decrease in ΦPSII photochemistry yields and in parameters of photochemical extinction in guard cells, suggesting that as-induced inhibition of guard cell photosynthesis may affect stomatal closure at high SA concentrations, as observed by Poo’r and Tari [53]. In addition, excessive reduction of ΦPSII was found to generate reactive oxygen species (ROS), as observed by high amounts of H_2_O_2_ in SA-treated leaves [28], suggesting an indirect secondary effect, regardless of water stress with SA. Notably, spatiotemporal mapping of leaf apoplastic ion and metabolite patterns provides valuable insights into SA mechanisms, especially under drought conditions. For example, Franzisky et al. [54] evaluated apoplastic abscisic acid (ABA) gradients in response to salinity and the unexpected accumulation of kaempferol glycosides in the leaf apoplast.

Plants in the irrigation treatment showed a reduction in NPQ, that is, a decrease in heat dissipation by xanthophylls (D) with SA concentrations of 0.5 and 1 mM. Plants under drought and rehydration conditions with SA applications of 1 mM showed higher NPQ values, demonstrating protection against damage resulting from water stress (Figure 4D,F), compared to plants with 1.5 mM. Higher D values were observed when 0.5 and 1.0 mM of SA were applied during rehydration (Figure 4F). A SA application of 0.5 mM also leads to higher energy accumulation in the photosystems (E) of irrigated plants (Figure 4G). Janda et al. [28] found that tobacco plants that received SA application had limited energy dissipation capacity. Lauterberg et al. [55] reinforce that NPQ contributed to protection against photodamage in plants subjected to water stress.

In the fluorescence extinction coefficients, qP and NPQ showed different responses concerning plants with and without SA under different water conditions (Figure 3C,D and Figure 4C,D), and qP was higher in rehydrated plants with 1.5 mM SA, suggesting that the activation of carboxylation enzymes was faster (Figure 4C). Higher qP values indicate that fluorescence extinction for photochemical processes was greater in stressed plants or that more energy was dissipated in primary processes of photochemical reactions [56] (Figure 3C). Higher or constant qP values, along with a decrease in quinone A, indicate increased participation of alternative electron sinks, such as photorespiration [57]. Thus, at the moment when qP of drought-stressed plants tends to reach the same level as that of rehydrated plants, there may be an increase in the participation of photorespiration in the fluorescence photosynthetic extinction.

Exogenous SA application acts as a potential inducer of the defense response, increasing hydrogen peroxide (H_2_O_2_) levels in plants [58], which was confirmed in the present study, in which plants rehydrated with 0.5 and 1.5 mM SA had higher H_2_O_2_ concentrations than irrigated plants (Figure 5B). In rehydration, higher H_2_O_2_ content was verified when an SA concentration of 0.5 mM was applied (Figure 5B). Together, these factors contribute to the activation and regulation of antioxidant enzymes (POD, APX, CAT, and SOD), maintaining the H_2_O_2_ content balance, and helping plants preserve their structural integrity and functionality when faced with the detrimental and destabilizing impacts caused by ROS generated during stressful conditions. It was also observed that plants subjected to drought and rehydration with SA applications of 0.5 mM showed greater photoprotective capacity (Figure 4D), acting as an important factor in mitigating the effects of water stress. Sahu et al. [59] observed that SA applications of 50 μM in wheat plants did not change the H_2_O_2_ content. In the same study, the authors also observed that the highest SA concentration (1000 µM) increased the H_2_O_2_ level. Janda et al. [28] reinforce that SA can cause oxidative stress to plants, partly due to H_2_O_2_ accumulation. Thus, it is crucial to determine the optimal SA doses, as excessively high doses can cause phytotoxicity and adverse effects on plant growth and development. Therefore, it is essential to avoid excessive doses and monitor the effects of SA application on plants.

Thus, the response of SA in pepper plants is dose-dependent, in which high SA concentrations can increase susceptibility to water stress and influence plant metabolism. It should be emphasized that the ideal SA dose may vary depending on the plant species, growth stage, stress type and intensity, as well as environmental conditions.

In general, SA concentrations of 1 mM allowed for the maintenance of mechanisms in plants subjected to drought and the recovery of plants from rehydration, as evidenced by *gs* data, effective quantum yield, excessive energy dissipation, and maximum quantum yield data. Additionally, SA application together with irrigation water can increase fresh and dry masses, photosynthesis, and activities of key antioxidant enzymes (Supplementary Tables S1 and S2, Figure 2, Figure 4 and Figure 5B). However, the complex interaction between SA, photosynthesis, and oxidative stress underscores the need for further in-depth studies to elucidate the underlying molecular mechanisms.

This study suggests that SA may act as an antagonist to ABA in regulating stomatal conductance in response to water stress, as observed by Ghahremani et al. [44] in the stomatal closure of pepper plants in response to water deficit conditions due to increased ABA content in leaf tissue. SA was introduced as an inhibitory factor for ABA-induced stomatal closure. It appears that balanced ABA and SA levels in plant leaves lead to an appropriate stomatal response to water stress. The increase in ABA synthesis rate induced by water stress can significantly alter the ABA to SA ratio in plant leaves. However, exogenous SA application can be considered an appropriate method to increase SA content in leaf tissues and balance the ABA to SA ratio. This interaction between SA and ABA highlights the complexity of hormonal signaling networks in plants and the importance of considering these interactions when designing stress management strategies.

In summary, the detailed discussion on the mechanisms of SA in plant response to water stress provides important insights into understanding the underlying physiological processes and developing practical approaches to improve plant tolerance to environmental stress. This article provides evidence of the role of SA in mitigating water stress in pepper plants. However, it is crucial to continue investigating these mechanisms in different plant species and environmental conditions to optimize the use of SA to increase the resilience of agricultural crops.

For practical applications, it is also necessary to address limitations such as concentration-dependent effects of the elicitor used, as well as environmental variations [60], including fluctuations in temperature, relative humidity, and vapor pressure deficit. These factors can significantly influence the treatment efficacy, potentially altering the plant’s response to the elicitor and the overall success of stress mitigation strategies. A deeper understanding of these variables is crucial for optimizing elicitor applications under diverse environmental conditions, thereby ensuring consistent and reliable outcomes in agricultural practices.

## 4. Material and Methods

### 4.1. Experiment Location

The experiment was carried out in a greenhouse at São Paulo State University, Campus of Botucatu, Brazil, using plastic bags filled with soil samples. The average temperature of the hottest month is 22.0 °C and that of the coldest month is 17.5 °C, with an average annual temperature of 21 °C. During the experiment, there was humidity and temperature control in the greenhouse.

The maximum, minimum, and average temperatures, respectively, were 27 °C, 18 °C, and 22 °C, and the light intensity recorded during the experiment was 450 µmol m^−2^ s^−1^.

### 4.2. Seedling Production, Soil Physical and Chemical Characteristics and Irrigation Monitoring

In the experiment, hybrid ‘Melina’ pepper (*Capsicum annuum* L.) seeds with determined growth were used. This variety produces large-sized fruits with reddish color, rectangular shape, predominantly four locules, thick walls, and high post-harvest performance, being developed by Sakata Seed South America*^®^*, Bragança Paulista—SP, Brazil. The hybrid ‘Melina’ pepper was chosen for the present study due to its suitability for cultivation in tropical regions and high acceptance among both producers and consumers in the area. Additionally, it stood out among the materials tested in preliminary trials.

The sowing of hybrid ‘Melina’ bell pepper (*C. annum* L.) was carried out in 128-cell polystyrene trays, and after 45 days of emergence, seedlings with 8 cm in height and six pairs of definitive leaves were transplanted to plastic bags with a volume of 500 mL, containing soil (Oxisol) as substrate, with the following chemical characteristics: pH: 5.8; P: 84.0 mg dm^−3^; K: 1.9 mmolc dm^−3^; Ca: 21 mmolc dm^−3^; Mg: 7.0 mmolc dm^−3^; Al^3+^: 0.8 mmolc dm^−3^; organic matter: 12 g dm^−3^; H^+^ + Al^3+^: 13.0 mmolc dm^−3^; S: 2.0 mg dm^−3^; B: 0.27 mg dm^−3^; Cu: 7.0 mg dm^−3^; Fe: 54.0 mg dm^−3^; Mn: 6.7 mg dm^−3^; Zn: 3.5 mg dm^−3^; SB (sum of bases): 30.0; CTC (cation exchange capacity): 42.0 mmolc dm^−3^; V (base saturation): 70%.

Daily irrigation was carried out for 13 days, keeping seedlings in plastic bags at field capacity. For this purpose, all bags were irrigated to maximum water holding capacity (100%) and kept without irrigation overnight to release excess water. During this period, the surfaces of bags were sealed with aluminum foil to prevent evaporation. The following morning, bags were weighed, and their masses were used at the maximum water holding capacity (100%). The weight of bags (g) throughout the experiment compared with their weights at maximum holding capacity (100%) was used to calculate the water content in the substrate (CAS) using the following formula: CAS = (MTreatment/M100%) × 100, according to Varone et al. [61]. This calculation allowed controlling and replenishing water to keep bags containing plants at their maximum holding capacity, which procedure was daily performed.

Plants were maintained for 13 days after transplanting for acclimatization, receiving irrigation as previously indicated. After this period, plants with a 58-day cycle were submitted to treatments.

### 4.3. Water Regime

Plants were subjected to three water regimes and were maintained at field capacity (irrigation), drought, and rehydration. Changes in field capacity were monitored according to Thameur et al. [62], using the soil water potential meter Psychrometer Model Dewpoint Potentiometer (WP4-T) (ADDIUM, METER Group).

Plants subjected to the water deficit treatment were not irrigated until leaves showed signs of wilting. When they showed lower water potential, stomatal conductance, and water percentage, wilting and leaf + 1 curling were observed.

**Figure 6:**
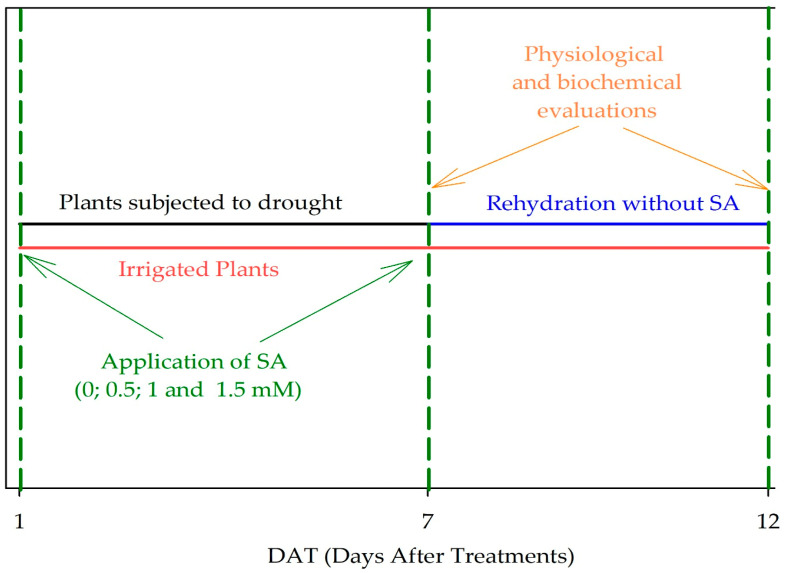
Pepper plants (*Capsicum annuum* L.) subjected to irrigation. Plants from the drought at 7 days after treatments (DAT) were subjected to rehydration. At times 1 and 7, SA was applied at concentrations of 0.5, 1, and 1.5 mM. Control treatment without the addition of SA (0).

The water deficit was maintained for seven days before plant evaluation. Rehydration treatment was applied after visual observation of leaf wilting, which occurred at 12 DAT when the substrate water content was 30% and the relative leaf water content was 61%. Plants were then evaluated five days after rehydration, when the soil had returned to field capacity (Figure 6).

### 4.4. Foliar Application of Salicylic Acid and Assessments

Plants were also subjected to salicylic acid (ortho-hydroxybenzoic acid) variation. Thus, they were submitted to irrigation, drought, and rehydration without the addition of a regulator (0) and with the addition of a regulator at concentrations of 0.5, 1, and 1.5 mM (Figure 6). These salicylic acid (SA) concentrations were chosen through preliminary tests and based on studies conducted under the same conditions [43], and according to research conducted by Nazar et al. [63] and Champa et al. [64].

SA was applied twice, half at 13 and half at 20 days after transplanting seedlings into plastic bags.

Solutions containing SA for each treatment were previously diluted in 10 mL of 90% ethanol and then dissolved in one liter of deionized water, according to Gonçalves et al. [43]. Leaves were sprayed with SA concentrations at 9:00 h with average temperatures close to 18 °C and relative humidity of 80%. For this purpose, a pressurized CO_2_ manual sprayer was used, with a “fan” nozzle at flow rates from 0.025 to 2500 gpm (0.09 to 9464 l/min) and a pressure of 4000 psi (276 bar), with the Agral^®^ commercial product as the spreading agent (Nonyl Fenoxy Poly (Ethyleneoxy) Ethanol), Paulínia—SP, Brazil, in the proportion of 800 µL per liter of SA solution.

Leaf relative water content, plant water potential, photosynthetic pigments, fluorescence of chlorophyll *a*, stomatal conductance, and hydrogen peroxide were evaluated at 7 and 12 days after treatments (DAT) (Figure 6).

### 4.5. Experimental Design

The experimental design was randomized blocks in a 3 × 4 factorial scheme, with three water regimes (irrigation, drought, and rehydration) and four SA concentrations (0; 0.5; 1; and 1.5 mM), prepared from the weighing of product from the Dynamics^®^ Company, PM 138.12 g (chemical formula C_7_H_6_O_3_) on an analytical scale with four replicates.

The irrigation regimes were established through preliminary tests and based on the study conducted by Mantoan et al. [65].

The experimental unit consisted of a plastic bag measuring 10 cm in diameter and 20 cm in height containing 1 plant.

In the first phase of the experiment, i.e., at 13 days after transplanting seedlings into bags, plants were subjected to the following water regimes: irrigation and drought, lasting for 7 DAT. In the second phase, plants were subjected to the following water regimes: irrigation and rehydration, lasting for 12 DAT (5 days after rehydration). These evaluation intervals of 7 and 12 DAT were established through preliminary tests and by monitoring stomatal conductance (mmol.m^−2^ s^−1^) throughout the day at intervals of one to two hours using a porometer (Decagon Devices), ADDIUM, METER Group, São José dos Campos—SP, Brazil.

### 4.6. Leaf Relative Water Content (RWC)

A leaf was taken from four plants per plot before, during, and after water stress to assess the leaf relative water content (RWC) of plants. For this purpose, duplicate areas (2 cm × 2 cm) of leaves from plants were weighed to determine fresh mass (FM). Immediately after weighing, samples were immersed in deionized water for four hours to determine turgid mass (TM). After this determination, samples were dried in a forced-air oven at 70 °C until reaching constant mass to determine dry mass (DM).

The mass values obtained were used in the formula proposed by Weatherley [66]: RWC = (MF − MS)/(MT − MS) × 100, to determine RWC.

### 4.7. Plant Water Potential (Ψw)

Plant water potential (Ψw) (kPa) was determined between 05:00 and 06:00 h in the morning, using a Scholander pressure pump [67] PMS Instrument Company, model 600, Piracicaba—SP, Brazil. Plant stem fragments were separated and immediately placed in the pressure chamber, where measurements were taken after immediate pressure application and liquid exudation [68].

### 4.8. Chlorophylls a and b and Total Carotenoid Content

To determine chlorophyll (*a* and *b*) and total carotenoid content, leaf discs from plants were collected and placed in test tubes containing 1 mL of dimethylformamide reagent. Test tubes were covered with aluminum foil and stored under refrigeration at 4 °C for 24 h, after which the entire solution volume was transferred to a cuvette, and readings were performed in a spectrophotometer at wavelengths of 480, 646.8, and 663.8 nm for the determination of chlorophyll *a* and *b* and total carotenoid content. Pigments were determined according to Moran. [69] and Lichtenthaler and Buschmann [70], expressed in µg cm^−2^.

Chlorophyll *a*: 12 × absorbance at 663.8 nm − 3.11 × absorbance at 646.8 nm.

Chlorophyll *b*: 20.78 × Absorbance at 646.8 nm − 4.88 × absorbance at 663.8 nm.

Total carotenoids: (1000 × absorbance a 480 − 1.12 × chlorophyll *a* − 34.07 × chlorophyll *b*)/245.

### 4.9. Stomatal Conductance

To evaluate stomatal conductance (mmol. m^−2^ s^−1^), a porometer (Decagon Devices) was used. Readings were performed on completely expanded leaves of the middle section of plants at 8:00 h, 10:00 h, 12:00 h, 14:00 h, and 16:00 h.

### 4.10. Chlorophyll a Fluorescence

Fluorescence was evaluated using a pulse-modulated fluorometer (JUNIOR-PAM, Walz^®^), Walz Teaching-PAM, São Paulo—SP, Brazil. Measurements were performed on irrigation, drought, and rehydration treatments.

Evaluations were performed at 12:00 h. Leaves were acclimatized for a period of 30 min in the dark with aluminum foil. Then, a saturation pulse of 10,000 μmol m^−2^s^−1^ of photosynthetically active photon flux density (DFFFA) with 0.6 s was applied to obtain Fm (maximum dark-adapted fluorescence) and Fm′ (maximum light-adapted fluorescence). In addition to maximum light-adapted leaf fluorescence, Fo′ (minimum light-adapted fluorescence) was also obtained. Between each saturation pulse, an actinic light pulse of 1150 μmol m^−2^ s^−1^ of DFFFA of 15 s duration was performed.

Using Fm′ and Fo′, the maximum quantum yield (Fv′/Fm′) was calculated, which reflects the ability of PSII to oxido-reduce the primary QA acceptor (quinone A) [71]. In addition, the following parameters were also calculated: effective quantum yield (ΦPSII), which corresponds to the energy fraction that is photochemically converted into photosystem II (PSII) [72]; photochemical quenching (qP), which reflects the photosynthetic carbon metabolism [73]; non-photochemical quenching (NPQ), which corresponds to photoprotection mechanisms [74]; and electron transport rate (ETR), which corresponds to the transport of electrons between photosystems, considering that 84% of light is absorbed by chlorophyll, with 50% of photons activating photosystem II chlorophyll and 50% photosystem I [75]. Maximum quantum yield (Fv′/Fm′), ΦPSII, qP, ETR, heat dissipation (D), energy dissipation (E), and NPQ determinations were represented by the mean values of two leaves.

### 4.11. Determination of the Hydrogen Peroxide (H_2_O_2_) Content

To determine the hydrogen peroxide (H_2_O_2_) content, leaf samples were collected at 9:00 h, and the methodology by Alexieva et al. [76] was adopted. Samples of 0.1–0.25 g were homogenized in liquid nitrogen, and 1 mL of 0.1% TCA was added, followed by vortexing. Subsequently, samples were centrifuged at 12,000× *g* for 15 min at 4 °C.

For the reaction, 0.5 mL of extract + 0.5 mL of 0.1 M phosphate buffer (pH 7.0) + 2.0 mL of 1 M KI were used, with extract and reaction buffers kept in the dark for 1 h. Readings were performed in a spectrophotometer at 390 nm, and the H_2_O_2_ concentration was calculated considering the curve (0–100 µM), measuring the ratio between H_2_O_2_ content and the dry mass of samples.

### 4.12. Statistical Analysis

Physiological and biochemical variables were submitted to normality and variance homogeneity analysis, respectively, using the Shapiro-Wilk and Bartlett’s tests. Subsequently, parametric tests such as one-way ANOVA at 1% and 5% probability were performed, and when significance was observed, the Tukey test at 5% significance level was adopted [77]. Analyses were conducted in the R platform with the assistance of the ExpDes.pt package [78].

## 5. Conclusions

Based on our findings, it was concluded that pepper (*Capsicum annuum* L.) plants are sensitive to water deficit, which affects stomatal conductance and chlorophyll *a* fluorescence. However, the application of salicylic acid (SA) at a concentration of 1.0 mM can mitigate the adverse effects of water stress by modulating physiological mechanisms, particularly in plants subjected to drought followed by rehydration.

The results indicate that SA treatment increased stomatal conductance and enhanced the electron transport rate, improving overall photosynthetic efficiency. In plants treated with 1.0 mM of SA, key parameters such as effective quantum yield, electron transport efficiency, and non-photochemical quenching behaved similarly to well-watered plants.

This study highlights the potential of SA as an environmentally safe and cost-effective management strategy to reduce drought-related damage in pepper plants. Future research should focus on metabolomic studies to further elucidate the genes involved in enhancing crop tolerance to water deficits.

## Data Availability

The data presented in this study are available on request from the corresponding author.

## Supplementary Materials

The following supporting information can be downloaded at: https://www.mdpi.com/article/10.3390/plants13192805/s1, Figure S1: Temperature (A), relative humidity (B) and air vapor pressure deficit (C); Table S1: Relative water content in leaves (%) of pepper plants (*Capsicum annuum* L.) throughout the day; Table S2: Plant water potential (kPa), chlorophyll *a*, *b* and carotenoid content (µg cm^-2^) of pepper plants (*Capsicum annuum* L.); Table S3: Analysis of variance for stomatal conductance (*gs*) in mmol m^-2^s^-1^ throughout the day of pepper plants (*Capsicum annuum* L.).
