# Supplementary material for: Effects of Salicylic Acid on Physiological Responses of Pepper Plants Pre-Subjected to Drought under Rehydration Conditions
Source: Plants (Basel). 2024 Oct 7;13(19):2805. doi: 10.3390/plants13192805 (PMC11479176; doi:10.3390/plants13192805)
Supplement: Supplementary file 1 [file plants-13-02805-s001.zip › plants-3195735-supplementary.pdf]

plants

Supplementary Materials

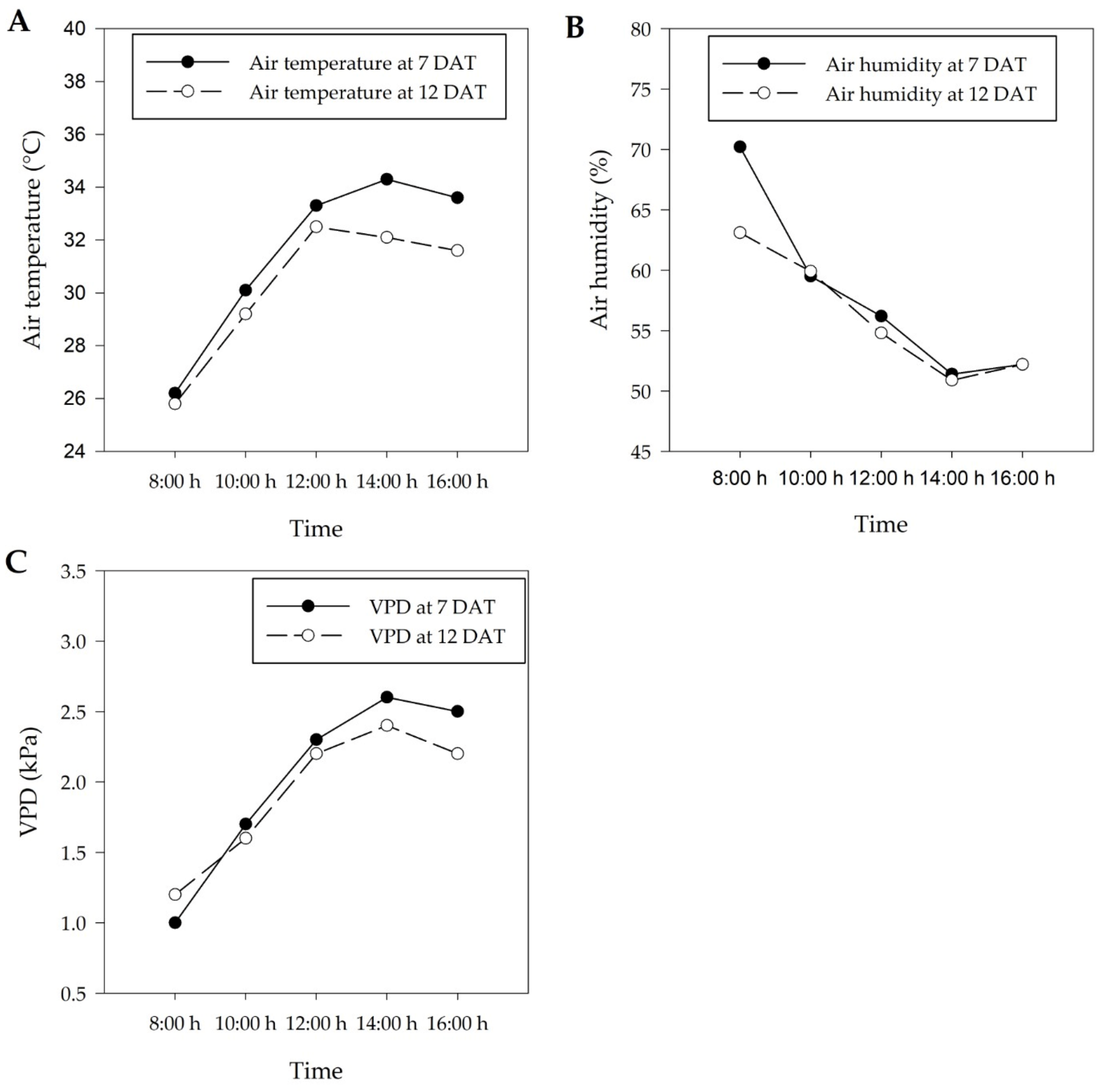

Figure S1. Temperature (A), relative humidity (B) and air vapor pressure deficit (C).

Table S1. Relative water content in leaves (%) of pepper plants (*Capsicum annuum* L.) throughout the day.

Treatment	8:00 h	10:00 h	12:00 h	14:00 h	16:00 h
7 days after treatments					
Irrigation (%)	74.0	70.4	74.2 a	70.1 a	66.6
Drought (%)	73.6	70.7	70.2 b	64.9 b	63.6
Significance					
Water Condition	n.s.	n.s.	P<0.01	P<0.05	n.s.
SA Concentration	n.s.	n.s.	n.s.	n.s.	n.s.
Water Condition X SA	n.s.	n.s.	n.s.	n.s.	n.s.
12 days after treatments (5 days after rehydration)					

Irrigation (%)	69.8	74.6	74.1	69.0	68.1
Rehydration (%)	68.8	74.4	73.8	68.8	65.1
Significance					
Water Condition	n.s.	n.s.	n.s.	n.s.	n.s.
SA Concentration	n.s.	n.s.	n.s.	n.s.	n.s.
Water Condition X SA	n.s.	n.s.	n.s.	n.s.	n.s.

Table S2. Plant water potential (kPa), chlorophyll *a*, *b* and carotenoid content (µg cm^{-2}) of pepper plants (*Capsicum annuum* L.).

7 days after treatments				
Treatment	Plant water potential	Chlorophyll *a*	Chlorophyll *b*	Carotenoids
Irrigation (%)	0.28	5.64	1.53	2.26
Drought (%)	0.29	5.62	1.53	2.43
0 mM	0.28	6.42 a	1.67	2.56 a
0.5 mM	0.27	5.44 ab	1.47	2.30 ab
1 mM	0.31	5.52 ab	1.56	2.35 ab
1.5 mM	0.29	5.13 b	1.43	2.17 b
Significance				
Water Condition	n.s.	n.s.	n.s.	n.s.
SA Concentration	n.s.	P<0.05	n.s.	P<0.05
Water Condition X SA	n.s.	n.s.	n.s.	n.s.
12 days after treatments (5 days after rehydration)				
Irrigation (%)	0.24	5.41	1.52	2.33
Rehydration (%)	0.27	5.90	1.58	2.49
0 Mm	0.31	6.22	1.91	2.52
0.5 mM	0.25	5.31	1.29	2.33
1 mM	0.22	5.48	1.50	2.37
1.5 mM	0.23	5.61	1.51	2.43
Significance				
Water Condition	n.s.	n.s.	n.s.	n.s.
SA Concentration	n.s.	n.s.	n.s.	n.s.
Water Condition X SA	n.s.	n.s.	n.s.	n.s.

Table S3. Analysis of variance for stomatal conductance (*gs*) in mmol $m^{-2}s^{-1}$ throughout the day of pepper plants (*Capsicum annuum* L.).

		Mean Square				
Sources of Variation	Degrees of Freedom	8:00 h	10:00 h	12:00 h	14:00 h	16:00 h
Water Condition	1	6.212^{ns}	9617.111^{ns}	379963.134^{**}	779298.095^{**}	67074.109^{**}
SA Concentration	3	171.034^{*}	6233.781^{ns}	66236.888^{**}	11223.943^{ns}	12554.731^{*}
Water Condition X SA	3	276.710^{**}	20249.511^{**}	15228.912^{ns}	16299.096^{ns}	12776.480^{*}
Residue	24	47.405	5022.954	11725.886	14237.689	2109.872
CV(%)		17.30	38.77	25.90	32.21	26.14

(^{ns}): not significant; (*) significant by 5% F test; (**) significant by 1% F test.
